# Structure/Function relationship and retinal ganglion cells counts to discriminate glaucomatous damages

**DOI:** 10.1186/s12886-015-0177-x

**Published:** 2015-12-29

**Authors:** Pietro Distante, Sara Lombardo, Alice Chandra Verticchio Vercellin, Marta Raimondi, Massimiliano Rolando, Carmine Tinelli, Giovanni Milano

**Affiliations:** University Eye Clinic, Fondazione IRCCS Policlinico San Matteo, Pavia, Italy; Department of Epidemiology and Biometry, Fondazione IRCCS Policlinico San Matteo, Pavia, Italy; University of Pavia, Pavia, Italy

**Keywords:** Glaucoma, Structure/function, Standard automated perimetry, Optical coherence tomography, Retinal ganglion cells count

## Abstract

**Background:**

Glaucoma is an optic neuropathy characterized by retinal ganglion cells (RGC) loss and retinal nerve fiber layer (RNFL) injury: this results in functional and morphological changes. The first can be observed by Standard Automated Perimetry (SAP), the second by Optic Coherence Tomography (OCT) that measures the RNFL and ganglion cell complex (GCC) thicknesses. Nevertheless, diagnosis of early glaucoma may be difficult. Recently, Medeiros et al. derived an empirical formula combining the measurement of structural and functional tests to provide an estimate of RGC. The aim of the current study is to analyse the correlation between RGC count, estimated by Medeiros’ formula, and the structural and functional parameters in patients examined for glaucoma and to evaluate SAP, OCT and RGC counts capability to discriminate the weight of the disease itself.

**Methods:**

Ninety four eyes of 50 consecutive patients clinically referring to glaucoma service of the Universitary Eye Clinic were submitted to a complete ophthalmic evaluation including SAP and Spectral Domain OCT (SD-OCT) of RNFL and macular GCC. Average thickness of RNFL and macular GCC, parameters Global Loss Volume (GLV) and Focal Loss Volume (FLV) over the entire GCC map were taken into account. Estimates of RGC were obtained with the help of a model already published by Medeiros et al. combining light sensitivities from SAP and retinal thickness from OCT. The RGC count was estimated in the entire visual field (central 24°) and in the GCC macular area and then compared with functional and morphological parameters applying Pearson’s correlation coefficient.

**Results:**

After the classification of the patients by the Glaucoma Staging System 2 of Brusini, we noticed a good correlation among the functional parameters considered, even if the Visual Field Index is unable to identify early glaucoma. An analogous result can be observed for structural data (RNFL and GCC). The correlation detected between functional and structural parameters was moderate. Great differences in RGC counts were found between groups at various stages of glaucoma. GLV showed highest level of correlation (r > −0.8) with RCG counts.

**Conclusions:**

Estimate circumpapillary and macular RGC counts can discriminate various stages of the disease and there is also a good/very good correlation with both functional and structural parameters. GLV could be used instead of RGC counts in clinical practice.

**Electronic supplementary material:**

The online version of this article (doi:10.1186/s12886-015-0177-x) contains supplementary material, which is available to authorized users.

## Background

Glaucoma is a term that encompasses a broad spectrum of diseases characterized by optic neuropathy with progressive and irreversible loss of Retinal Ganglion Cells (RGC), Optic Nerve Head (ONH) damage and Retinal Nerve Fiber Layer (RNFL) injury [1–3] resulting in morphological and functional changes. Early diagnosis and treatment are important in maintaining visual function and preventing vision loss [4]. Intra-Ocular Pressure (IOP) is the main risk factor and the only treatable one.

Standard Automated Perimetry (SAP), particularly the 24–2 Swedish Interactive Threshold Algorithm (SITA) standard strategy, has become the clinical gold standard for diagnosis and monitoring of patients with glaucoma [5].

Primary Open-Angle Glaucoma (POAG) is an optic neuropathy appearing in absence of other ocular diseases. POAG affects both eyes in an asymmetric way and it is usually asymptomatic until the later stages. Therefore, the diagnosis of early glaucoma may be difficult: IOP is not necessarily related to ONH damage, structural ONH or RNFL alterations can precede detectable changes by SAP [6, 7] or functional deterioration can be present without measurable changes in structural tests currently available [8–10].

Several instruments are available today in order to perform a structural ONH evaluation, such as Heidelberg Retina Tomograph (HRT), scanning laser polarimetry GDx and Optical Coherence Tomography (OCT). Spectral Domain OCT (SD-OCT) is able to map retinal substructures [11], such as RNFL and the ganglion cell complex (GCC). GCC is composed by the three innermost retinal layers (nerve fiber layer, ganglion cell layer and inner plexiform layer) containing axons, cell bodies and dendrites of the RGC. Recently, Medeiros et al. [12], on the base of experimental studies in monkeys, derived an empirical model combining the measurement of structural (average RNFL thickness) and functional tests (visual field sensitivity, mean defect) to provide an estimate of RGC in glaucoma patients.

The aim of the current study is to analyse the correlation between RGC count, estimated by Medeiros’ formula, and structural and functional parameters, obtained by SD-OCT and SAP, in patients examined for glaucoma and to evaluate SAP, OCT and RGC counts capability to discriminate the weight of the disease itself.

## Methods

### Subjects

Fifty glaucoma patients or glaucoma suspects (94 eyes) were consecutively recruited at the University Eye Clinic, IRCCS San Matteo Hospital, Pavia, Italy. The research adhered to the tenets of the Declaration of Helsinki. Each patient signed an informed consent that has been approved from the Institutional Human Experimentation Committee. We considered data concerning a single eye for six of these patients, because of the absence of fixation of the contra lateral eye resulting from advanced glaucoma so that SAP and OCT could not be performed. Each study participant underwent a complete ophthalmic examination including clinical history, best-corrected visual acuity (BCVA) ≥7/10 (if less than 7/10 the patient was excluded from the study), slit lamp biomicroscopy of the anterior segment, gonioscopy, Goldmann applanation tonometry, central cornea ultrasonic pachimetry and ophthalmoscopy of the posterior segment using a +90 D lens. IOP and eye drop therapy were not part of the inclusion/exclusion criteria. Participants were excluded if they had any retinal disease, non glaucomatous optic neuropathy or any significant coexisting systemic disease with possible ocular involvement, such as diabetes mellitus.

### Standard automated perimetry (SAP)

All the participants were also subjected to functional and structural evaluation of the optic nerve by SAP (24–2 SITA-Standard of Humphrey Field Analyzer II, Carl Zeiss MeditecInc) and SD-OCT (iVue, Optovue, Inc., Fremont, CA).

Regarding SAP, global indices Mean Defect (MD), Pattern Standard Deviation (PSD) and Visual Field Index (VFI) were taken into account. All patients were well trained to SAP with more than one reliable visual field test in the past. All the visual field considered were reliable with fixation losses <20 % and false positive and negative errors <15 % and were performed only once on the examination day.

Glaucoma severity was staged by MD and PSD, according to GSS2 of Brusini and ranging from Stage 0 (normal) to Stage 5 (advanced). Three groups were formed: group 0 (Stage 0 and borderline), group 1 (Stage 1 and Stage 2) and group 2 (Stage 3, Stage 4 and Stage 5), as shown in Table 1. MD was also calculated as the arithmetic mean of the 16 central points of the visual field corresponding to the GCC area in the Total Deviation graph.

**Table 1:** Summary of considered parameters and results

	Total	Group 0 (Stages 0-border)	Group 1 (Stages 1–2)	Group 2 (Stages 3–4–5)
Number of eyes	94	49	21	24
Age^b^	66.38 (11.69)	64.08 (12.35)	69.38 (8.89)	68.46 (11.9)
MD^a^	−1.815 (−6.02- -0.3)	−0.37 (−1.02–0.11)	−3.21 (−4.17 ­ -2.71)	−13.42 (−21.98 ­ -9.70)
PSD^a^	2.315 (1.61–-7.2)	1.65 (1.52–-1.86)	3.02 (2.48–-3.75)	9.45 (7.86–-11.36)
VFI^b^	87.26 (21.72)	98.69 (1.25)	94.14 (3.35)	57.88 (25.9)
RNFL av. (μm)^b^	86.19 (16.44)	94.98 (10.78)	85.9 (17.03)	68.5 (10.39)
RNFL av. sup. (μm)^b^	86.22 (16.09)	95 (11.28)	84.81 (14.58)	69.54 (11.85)
RNFL av. inf.(μm)^b^	86.18 (19.03)	95.02 (13.12)	86.86 (22.45)	67.54 (11.73)
RNFL av.temp. (μm)^b^	87.2 (18.63)	98.96 (14.17)	78.42 (15.37)	70.86 (11.62)
GCC av. Total (μm)^b^	82.95 (12.69)	89.47 (8.26)	81.9 (12.89)	70.54 (10.57)
GCC av. sup. (μm)^b^	83.03 (12.8)	89.39 (8.66)	82 (12.51)	70.96 (11.42)
GCC av. inf. (μm)^b^	82.85 (13.43)	89.57 (8.8)	81.81 (14.22)	70.04 (11.1)
RGC count^b^	723278.3 (297940.9)	935795.4 (155703.3)	680545.1 (149734)	326780.8 (164051.3)
RGC count GCC^b^	700102.9 (300675.8)	909314.2 (173798.6)	662358.6 (139205.6)	305989.2 (170404.9)
GLV (%)^b^	14.46 (11.08)	7.96 (6.47)	16.16 (9.02)	26.25 (10.03)
FLV (%)^a^	2.719 (0.64–8.12)	1.108 (0.49–2.16)	5.454 (1.20–7.53)	9.408 (7.40–11.35)

Data with (^a^) are expressed as Median and Interquartile Range, IQR (showed in parenthesis). Data with (^b^) are expressed as Media and Stardard Deviation, SD (showed in parenthesis). Thicknesses: RNFL average (RNFL av.); RNFL average superior (RNFL av. sup.); RNFL average inferior (RNFL av. inf.); RNFL average temporal (RNFL av. temp.); GCC average (GCC av. Total); GCC average superior (GCC av. sup.); GCC average inferior (GCC av. inf.). Circumpapillary retinal ganglion cells count (RGC count). Macular retinal ganglion cells count (RGC count GCC). Global loss volume (GLV). Focal Loss Volume (FLV)

### Optical coherence tomography (OCT) and data processing

The eyes of the participants were scanned with iVue SD-OCT system (Optovue, Inc., Fremont, CA) which acquires 26000 axial scans (a-scans) per second and has a 5-μm depth resolution. ONH and GCC scan patterns were used.

The ONH scan protocol measures circumpapillary RNFL thickness by calculating data along a circle of 3.45 mm in diameter around the optic disc: this circle is created by a scan pattern made up of 13 concentric circular scans ranging from 1.3 to 4.9 mm in diameter with 0.3 mm interval and 12 radial scans 3.40 mm in length (455 A-scans each), all centered on the optic disc. This scan configuration provides 14141 A-scans in 0.55 s. Areas between A-scans are interpolated. A polar RNFL thickness map and various parameters that describe the optic disc are provided. RNFL thickness measurements were obtained for the 3.45 mm diameter ring.

Average RNFL and sectorial (superior: *RNFL av. sup.*; inferior: *RNFL av. inf.*; temporal: *RNFL av. temp.*) thicknesses, provided by OCT, were considered. Average temporal RNFL thickness refers to the arithmetic mean between all temporal sectors.

Two recent studies [13, 14] have found that glaucoma diagnostic accuracy could be improved if macular measurements by OCT are focused on the inner retinal layers. Three innermost retinal layers are preferentially affected by glaucoma: the nerve fibre, ganglion cell, and inner plexiform layers, which contain, respectively, the axons, cell bodies, and dendrites of the ganglion cells. Therefore, we refer to the combination of these three layers as the GCC.

The macular GCC scan protocol using SD-OCT iVue consists of scan 15000 points in a 7 mm square area within 0.6 s by using one horizontal line and 15 vertical lines at 0.5 mm intervals. The scans are centred 0.75 mm temporarily to the fovea to improve the coverage of temporal macula: these are processed automatically to provide a map of ganglion cell complex. Average GCC total (*GCC av. Total*), superior (*GCC av. sup.)* and inferior (*GCC av. inf.)* thicknesses were considered.

Global loss volume (GLV) and focal loss volume (FLV) are two new parameters for the GCC scan in the 4.0 software. GLV measures the average amount of GCC loss over the entire GCC map, based on the fractional deviation (FD) map. This value is the sum of individual deviation values at each pixel where the FD map value is <0, which is then divided by the total area to give an average percentage loss of GCC thickness. FLV measures the average amount of focal loss over the entire GCC map and is based on both the FD map and the pattern deviation (PD) map. The PD map is determined by first calculating the individual pattern maps from all individuals in the normative database.

Regarding GCC, scan total GCC average, superior and inferior GCC, FLV and GLV were taken into account in the current study.

**Fig. 1:**
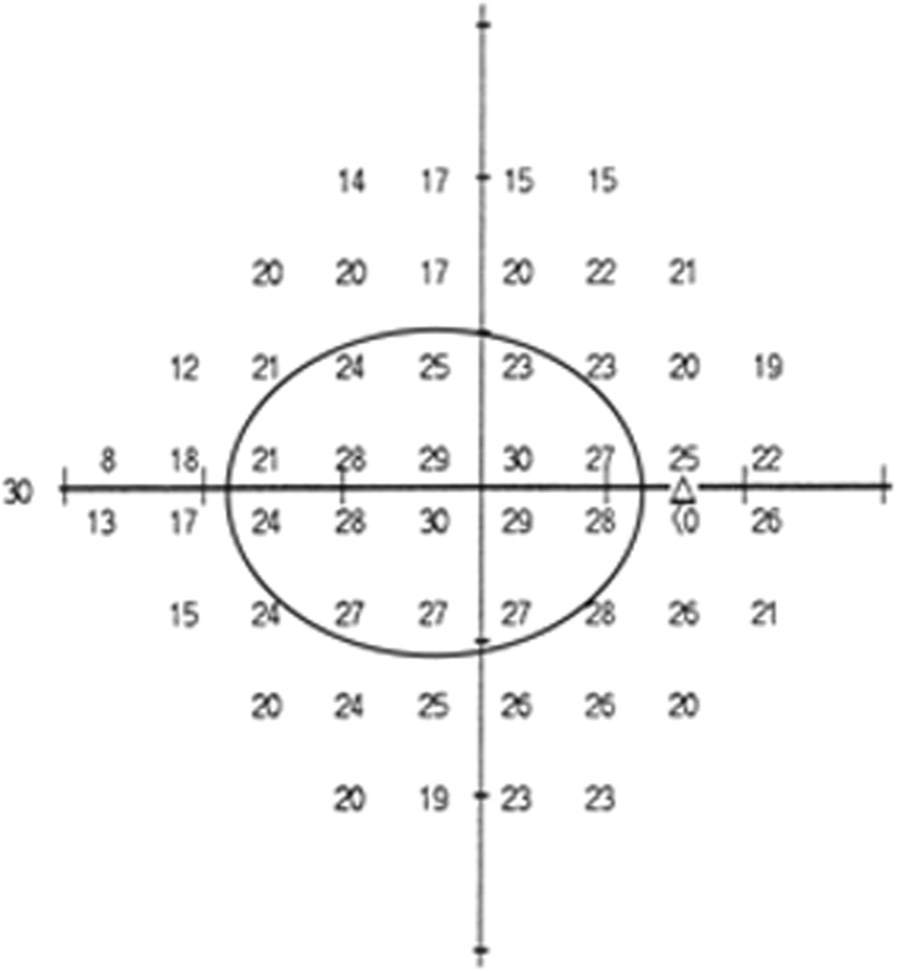
16 central threshold sensitive points of the visual field corresponding to GCC area

Algorithms processed by Medeiros et al. [12] were then applied, obtaining a total estimate of retinal ganglion cells (RCG count) and a relative one (RGC count GCC), taking into account only the 16 central values of the map of the thresholds in the visual field (Fig. 1) and corresponding to GCC area.

The first OCT test with a “Good” Scan Quality Index (≥40) was taken into account.

### Estimate the number of RGC

Estimate of RGC count was obtained from SAP and OCT considering Medeiros’ empirical models [12], and a weighted average was used to obtain a final estimate of the number of RCGs for each eye in circumpapillary and macular region.

Below, it is showed what formulas were used to estimate the number of RGC bodies in an area of the retina corresponding to a specific SAP test field location at eccentricity (*ec*) with sensitivity (*s*) in decibels. In these formulas, *m* and *b* represent the slope and the intercept, respectively, of the liner function relating ganglion cell quantity (*gc*) measured in decibels to the visual field sensitivity (*s*) in decibels at a given eccentricity (*ec*).

A SAP-derived estimate of the total number of RGC (*SAPrgc*) was obtained by adding the estimates from all locations in the visual field. For an estimate of RGC in macular region, we only considered sensitivities of 16 central points of the visual field (Fig. 1), using, into Medeiros’ formula, the MD calculated in the 16 central point of the Total Deviation map.

$$ m = \left[0,054\times \left(ec\times 1,32\right)\right]+0,9 $$

$$ b=\left[-1,5\times \left(ec\times 1,32\right)\right]\hbox{--} 14,8 $$

$$ gc = \left\{\left[\left(s\hbox{--} 1\right)\hbox{--} b\right]/m\right\}+4,7 $$

$$ SAPrgc=\varSigma \kern0.2em 10\wedge \left(gc\times 0,1\right) $$

The structural part of the model consisted of estimating the number of RGC axons from RNFL and GCC thickness measurement obtained by OCT (*OCTrgc)*. Below, formulas used are showed: *d* corresponds to the axonal density (axons/μm [2]) and *c* is a correction factor for the severity of the disease to take into account remodeling of the RNFL axonal and non-axonal composition. The average RNFL and GCC thicknesses correspond to the 360° measures automatically calculated by OCT software.

$$ d=\left(-0,007\times age\right)+1,4 $$

$$ c=\left(-0,26\times MD\right)+0,12 $$

$$ a= average\; RNFL\; thickness\times 10870\times d $$

$$ OCTrgc=10\wedge \left\{\left[ log(a)\times 10-c\right]\times 0,1\right\} $$

These calculations provide an estimate of the numbers of RGCs from two sources (one functional and one structural), so a combined measure was developed by Medeiros et al. [12] to combine *SAPrgc* and *OCTrgc*. Because clinical perimetry and imaging test are inversely related to disease severity, a weighted scale was used:

$$ Combined\ RGC\  count = \left(1 + MD/30\right) \times OCTrgc + \left( - MD/30\right) \times SAPrgc $$

RGCs were counted in the circumpapillary region using the average RNFL thickness (*RGC count*) and “average RNFL thickness” was replaced with the average GCC thickness to obtain another estimate of RGC count in the macular region (*RGC count GCC*).

### Statistical analysis

If quantitative data were normally distributed mean and standard deviation (SD) was used to summarize results; if data were not normally distributed median and interquartile ranges (IQR) was used. Qualitative data were described as frequencies and percentages. For quantitative variables, comparisons among three groups were performed with the ANOVA test or with One-way analysis of variance by ranks (Kruskal-Wallis Test) and post-hoc tests were performed to correct for multiple comparisons. Chi-square test or Fisher’s exact tests were used to compare qualitative variables. Correlations between quantitative variables were evaluated with Pearson’s *r coefficient*. The strength of correlation was defined as “very poor” (r < 20), “poor” (0.21 < r < 0.40), “moderate” (0.21 < r < 0.60), “good” (0.61 < r < 0.80) or “very good” (0.81 < r < 1). All reported *p*-values were two-sided. Differences were considered significant when the two-sided *p* value was <0.01. All analyses were carried out with the STATA software (vers: 13, Stata Corporation, College Station, 2013, Texas, USA).

To summarize, parameters we chose to correlate are: age, Mean Deviation (*MD*), Pattern Standard Deviation (*PSD*), Visual Field Index (*VFI*), Global Loss Volume (*GLV*), Focal Loss Volume (*FLV*), average RNFL thickness (*RNFL av.*), RNFL superior thickness (*RNFL av. sup.*), RNFL inferior thickness (*RNFL av. inf.*), RNFL temporal thickness (*RNFL av. temp.*), GCC average thickness (*GCC av.Total*), GCC superior thickness (*GCC av. sup.)* and GCC inferior thickness (*GCC av. inf.)*. To these, we also added *RGC count* and *RGC count GCC* obtained using Medeiros’ algorithm.

## Results

The study included 94 eyes of 50 patients (M/F : 27/23) with SAP and iVue SD-OCT scans available. Six eyes were previously excluded owing to their inability to look fixedly. According to GSS 2, the 94 eyes were classified into stage 0 (39 eyes), borderline (10 eyes), stage 1 (14 eyes), stage 2 (7 eyes), stage 3 (7 eyes), stage 4 (8 eyes) and stage 5 (9 eyes). Three groups were formed: group 0 (stage 0 + borderline; 46 eyes), group 1 (stage 1 + stage 2; 19 eyes) and group 2 (stage 3 + stage 4 + stage 5; 24 eyes).

The patients were not uniformly distributed within the different groups (Table 1): 70 of the 94 eyes were normal or affected by early glaucoma. This sample was representative for an initial glaucomatous damage, which was related to perimetric indices, as expected, but also to morphometric data provided by OCT.

Table 2 showed statistical significance values of all considered parameters comparing groups 0, 1 and 2. MD and PSD derived from SAP always showed a statistically significant difference (*p* < 0.01) in the comparison between groups. Nevertheless, VFI was not statistically significant (*p* = 0.563) comparing groups 0 and 1: this could demonstrate that not all SAP parameters, considered separately, are always so sensitive in discriminating healthy subjects from those with early/moderate perimetric defects. As demonstrated by Brusini's GSS2, these parameters should be considered in couple at least (and not individually) to discriminate various stage of glaucoma.

**Table 2:** Statistical significances and ability of the analyzed parameters to discriminate between different stages of disease

	*p* values		
	Groups 0–1	Groups 1–2	Groups 0–2
MD	<0.01	<0.01	<0.01
PSD	<0.01	<0.01	<0.01
VFI	0.563	<0.01	<0.01
RNFL av.	0.020	<0.01	<0.01
RNFL av. sup.	<0.01	<0.01	<0.01
RNFL av. inf.	0.134	<0.01	<0.01
RNFL av.temp.	<0.01	0.214	<0.01
GCC av. Total	0.010	<0.01	<0.01
GCC av. sup.	0.022	<0.01	<0.01
GCC av. inf.	0.021	<0.01	<0.01
RGC count	<0.01	<0.01	<0.01
RGC count GCC	<0.01	<0.01	<0.01
GLV	<0.01	<0.01	<0.01
FLV	<0.01	<0.01	<0.01

**Fig. 2:**
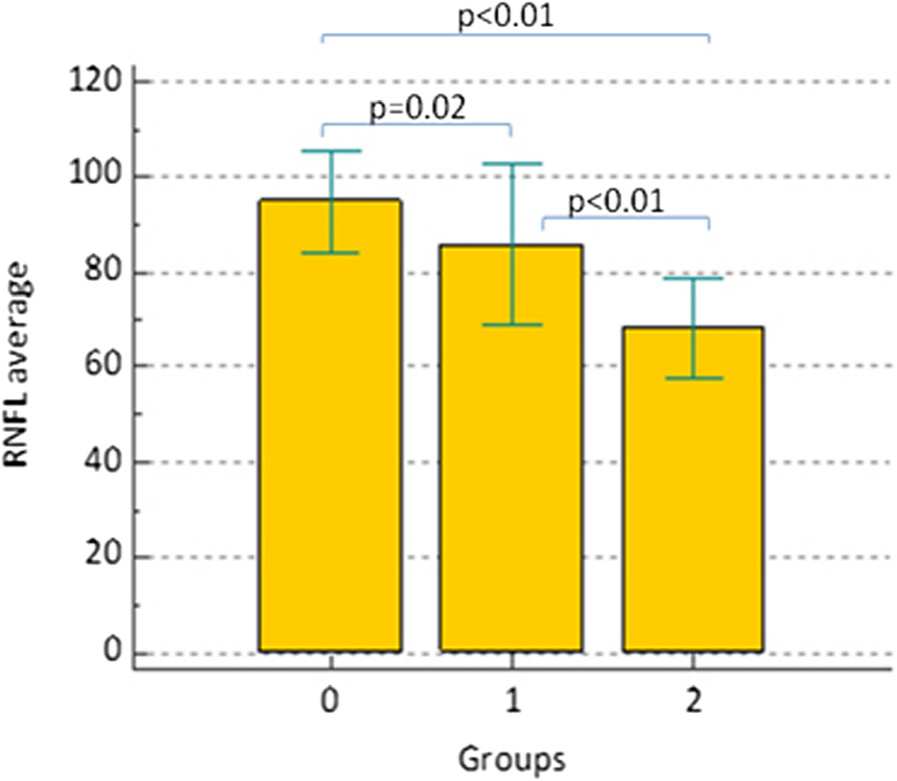
RNFL average thickness within three groups. Comments to Fig. 2: Error bars represent Standard Deviation (SD). Horizontal bars indicate *p* value comparing two groups each time

**Fig. 3:**
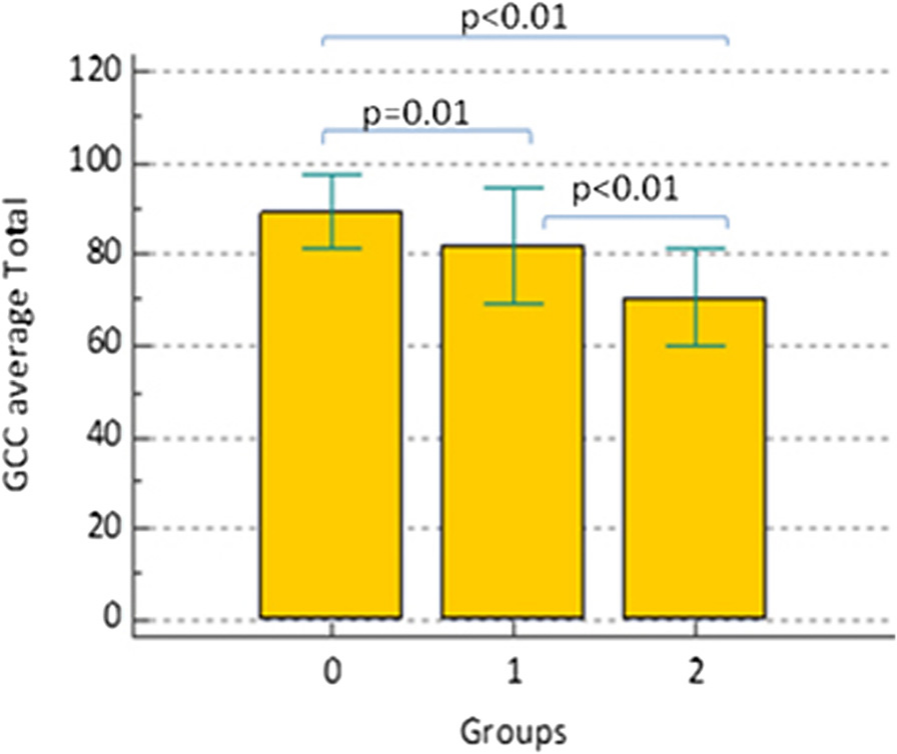
GCC average (GCC av.) thickness within three groups. Comments to Fig. 3: Error bars represent Standard Deviation (SD). Horizontal bars indicate *p* value comparing two groups each time

**Fig. 4:**
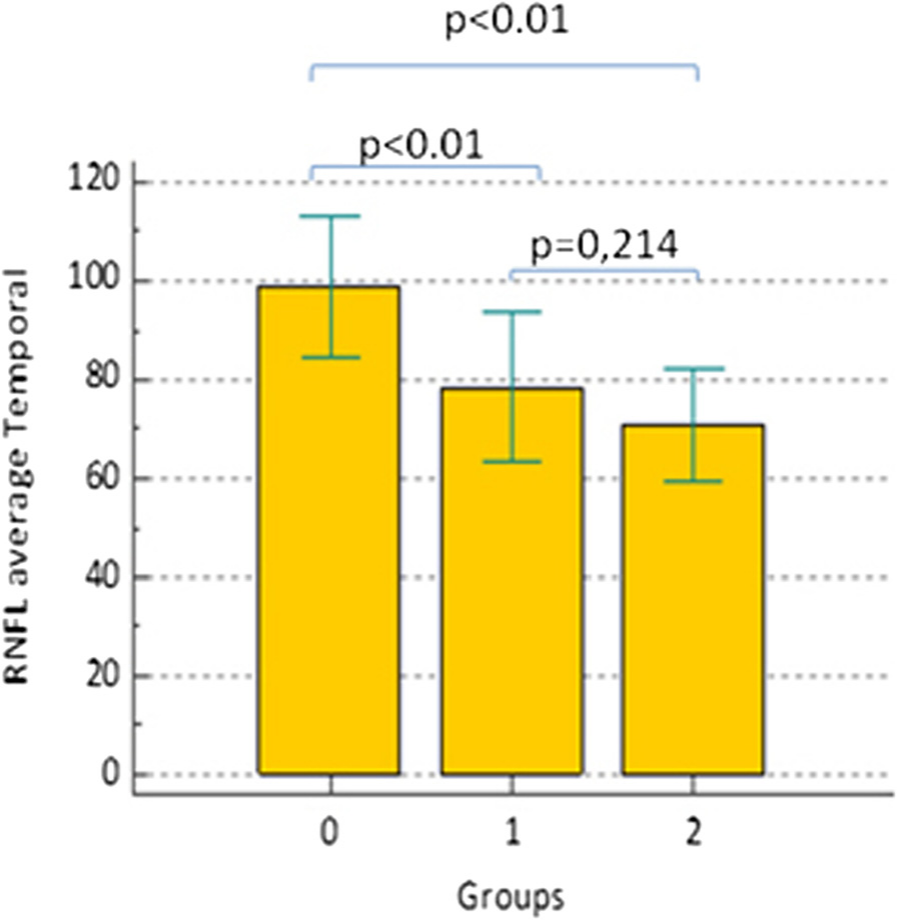
RNFL average temporal (RNFL av. temp.) thickness within three groups. Comments to Fig. 4: Error bars represent Standard Deviation (SD). Horizontal bars indicate *p* value comparing two groups each time

A similar pattern can be observed for circumpapillary and macular structural parameters: both total and sectorial RNFL and GCC thicknesses (Figs. 2 and 3) showed a *p* > 0.01 in comparison between group 0 and group 1, as it can been observed in Table 2. Two exceptions were represented by superior RNFL average and temporal RNFL average (*p* < 0.01): in particular, as it could be seen in Fig. 4, temporal RNFL average thickness showed a significant decrease comparing group 0 (98.96 μm; SD 14.17 μm) and group 1 (78.42 μm; SD 15.37 μm).

**Fig. 5:**
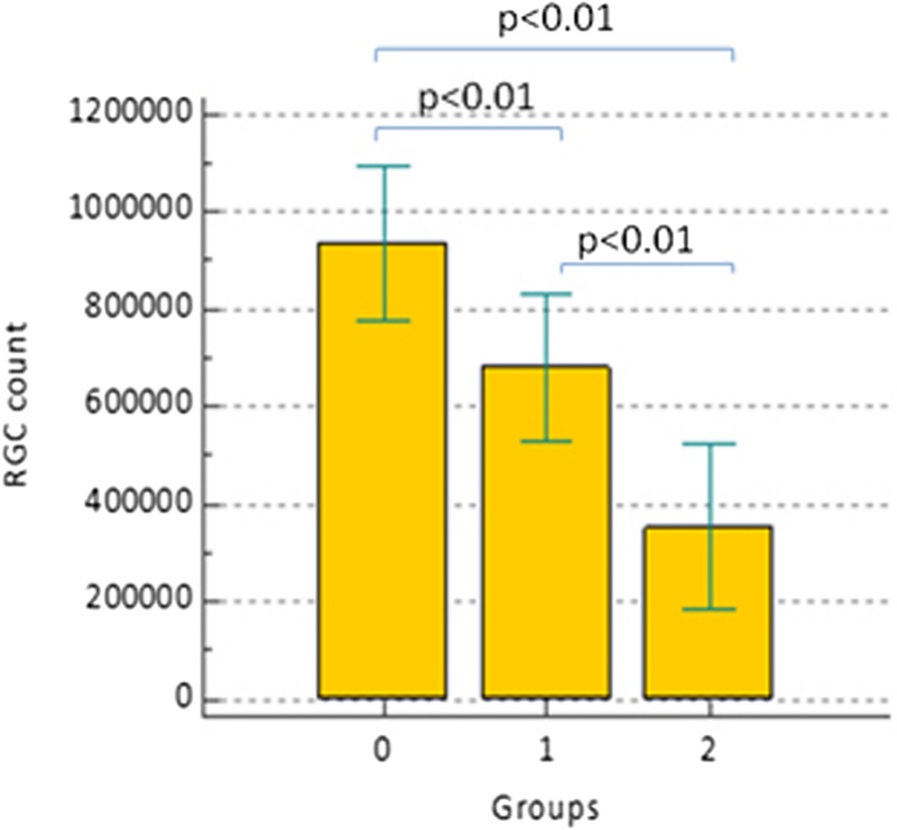
Estimate circumpapillary retinal ganglion cells count (RGC count) within three groups. Comments to Fig. 5: Error bars represent Standard Deviation (SD). Horizontal bars indicate *p* value comparing two groups each time

**Fig. 6:**
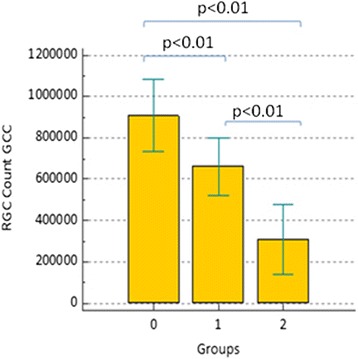
Estimate macular retinal ganglion cells count (RGC count GCC) within three groups. Comments to Fig. 6: Error bars represent Standard Deviation (SD). Horizontal bars indicate *p* value comparing two groups each time

On the contrary, estimate of retinal ganglion cells count, obtained applying Medeiros algorithms in circumpapillary and macular regions, seems to be able to discriminate patients at different stages of neuropathy. More in detail, as shown in Figs. 5–6, recorded circumpapillary RGC count (IQR) in group 0 was 935795.4 (155703.3), in group 1 was 680545.1 (149734) while in group 2 was 326780.8 (164051.3). On the other hand, macular RGC count (IQR) was 909314.2 (173798.6) in group 0, 662358.6 (139205.6) in group 1 and 305989.2 (170404.9) in group 2 (Fig. 3). Comparing these three groups, we always found *p* < 0.01 for both RGC count and RGC count GCC.

Considering Pearson’s correlation coefficient (r), there is no relation between SAP/OCT and the age of patients, so that our results are not influenced by this parameter (Table 3): in fact, no one of the considered parameters showed a statistically significant age-related difference. As expected, Pearson’s coefficient showed a good or very good correlation (r always >0.61) between parameters provided by the same instruments (SAP or OCT), but also between circumpapillary RNFL thickness and the inner macular retina (GCC), both total (r = 0.85) and sectorial superior/inferior (*r* = 0.78 and *r* = 0.87 respectively) (Table 3). Nevertheless, there is a substantially moderate correlation between structural and functional parameters, as shown in Table 3. Good and very good correlations (r always ≥0.61) can be observed comparing both functional and structural indices with RGC count both in the central macular area and paracentral one which correspond to the 24–2 visual field.

**Table 3:** Correlation strength (Pearson coefficient) between considered parameters

	Age	MD	PSD	VFI	RNFL average	RNFL av. sup.	RNFL av. inf.	RNFL av. temp.	GCC av. Total	GCC av. sup.	GCC av. inf.	RGC count	RGCcount GCC
Age	1.00												
MD	−0.09*	1.00											
PSD	0.16*	−0.79	1.00										
VFI	−0.09*	0.98	−0.76	1.00									
RNFL av.	−0.19*	0.60	−0.64	0.59	1.00								
RNFL av. sup.	−0.20*	0.60	−0.63	0.58	0.92	1.00							
RNFL av. inf.	−0.15*	0.54	−0.58	0.53	0.95	0.75	1.00						
RNFL av. temp.	−0.19*	0.55	−0.58	0.51	0.81	0.82	0.71	1.00					
GCC av. Total	−0.20*	0.60	−0.62	0.58	0.85	0.86	0.75	0.82	1.00				
GCC av. sup.	−0.21*	0.57	−0.59	0.55	0.78	0.85	0.63	0.78	0.96	1.00			
GCC av. inf.	−0.18*	0.58	−0.60	0.57	0.87	0.80	0.81	0.80	0.97	0.86	1.00		
RGCcount	−0.40	0.88	−0.81	0.82	0.81	0.79	0.74	0.65	0.76	0.73	0.75	1.00	
RGC count GCC	−0.40	0.85	−0.80	0.80	0.74	0.75	0.65	0.74	0.78	0.76	0.75	0.96	1.00
FLV	0.19*	−0.67	0.70	−0.66	−0.80	−0.79	−0.71	−0.76	−0.78	−0.76	−0.74	−0.79	−0.79
GLV	0.21*	−0.66	0.67	−0.64	−0.83	−0.85	−0.72	−0.83	−0.97	−0.94	−0.92	−0.81	−0.82

(*) Indicates that the correlation between those two parameters is not statistically significant (*p* > 0.01). In all the other cases without (*), *p* < 0.01

There is a good inverse correlation between FLV and the two RCG counts (*r* = −0.79); likewise, GLV is in a very good inverse relation with RGC count (*r* = −0.81) and RGC count GCC (*r* = −0.82).

## Discussion

Glaucoma is an optic neuropathy characterized by slow, progressive and irreversible loss of retinal ganglion cells and nerve fibres. It has been supposed that neuropathy could be present before cellular loss is detectable [15].

SAP is nowadays considered the clinical standard for diagnosis and follow-up of glaucoma, even if functional changes can be observed only after a great loss of RGCs and nerve fibers [16].

OCT is an imaging technique capable of providing “optical biopsies” of biologic tissue. The operation of OCT is based on the principle of low coherence interferometry: the distances and sizes of different structures in the eye are determined by measuring the “echo” time it takes for light to be back-scattered from different structures at various axial distances. SD-OCT allows fast and precise measurement of retinal thickness as well as visualization of intraretinal layers: in this way, the study of tissues and structures injured by glaucoma (RNFL and RGC) is certainly useful in diagnosis and treatment of the disease. In particular, RGC are evaluated in the macular region as GCC, a 7 mm squared area in which there could be a third of the whole number of RGC with their axons and dendrites [17]. The power of each OCT algorithm of analysis in detecting the smallest RGC and nerve fibres loss is extremely important for early neuropathy diagnosis and its changes during follow-up. Nevertheless, the use of computer algorithms to measure nerve fibre layer or macular thickness introduces segmentation error in cases where retinal layers are misidentified by computer software: OCT results can be negatively influenced by measurement of non-neuronal structures (as vessels and glial cells) contributing to retinal thickness. It is well known that structural tests as OCT are not so sensitive in pointing out small thickness variations in advanced glaucoma, when neuronal structures are already reduced [18, 19].

Medeiros proposes an estimate RGC count [12] based on SAP and OCT, and it seems to be well related to experimental glaucoma models in animals [12, 20, 21]. Moreover, it has been proved that Medeiros’ algorithm is able to characterize various stages of disease [16] and to recognize early glaucoma [22]. In all mentioned cases, it is referred to circumpapillary RNFL thickness.

The aim of the study is to analyze the strength of correlation between RGC counts estimated applying Medeiros’ formula, structural parameters obtained by SD-OCT and SAP indices in patients examined for glaucoma and their ability to discriminate different stages of the disease.

There is just a moderate correlation between functional and structural parameters (*r* = 0.5/0.6), confirming data in literature, but it is certainly interesting because of all the differences between SAP and OCT. Standard automated perimetry evaluates differential light sensitivities, measured in logarithmic scale (dB), and patient compliance is required for a good outcome and reliability of the test. On the other hand, performing optical coherence tomography needs first of all a skilful operator; moreover, thicknesses measurement (expressed in μm) depends from the instrument ability to recognize how all the different retinal structures reflect light.

A promising result has been obtained using Medeiros’ algorithm to estimate RGC in circumpapillary and macular region: both of them are useful to discriminate between the three groups of patients and they correlate in a very good way with structural and functional parameters. These results should be explained considering that Medeiros’ formula takes into account data coming from functional and structural tests. Moreover, each patient has different characteristics as age, stage of disease, neuronal and non-neuronal structures, differential light sensitivities. All these features are directly or indirectly involved in RGCs number determination, because some of them are considered within Medeiros formulas, while others (as non-neuronal structures) influence thicknesses measured by OCT.

Nowadays, glaucoma patients evaluation is based on the Visual Field and Spectral Domain OCT examinations. These tests are performed separately and their results are used by the ophthalmologist for glaucoma diagnosis and therapeutic choice. Several studies in the past demonstrated that correlation between functional and structural parameters is just moderate. Moreover, glaucoma specialists also take into account other data (age, life expectancy, severity of disease, compliance to therapy, neuropathy progression rate, risk factor, eccentricity, defect position in the visual field, etc.) that are left to a personal and subjective evaluation. Clinical reasoning is based on the combination of all these parameters (included Visual Field and OCT data): they are joined in a medical process that gives to the ophthalmologist the possibility to decide how to treat the disease. Considering glaucoma as an optic neuropathy with progressive retinal ganglion cells loss we can’t absolutely leave out the RGCs number from our clinical reasoning.

Unfortunately, a direct estimate of this number is not possible (it requires a retinal biopsy), so that we must be satisfied with an indirect evaluation. Medeiros’s formulas to estimate retinal ganglion cells number represent not only an interesting combination of functional and structural parameters, but also a method to take into account all other data (eccentricity, age, differential light sensitivity, axonal density, etc.) in an objective way: just a number to summarize a wide and partially personal clinical reasoning. Retinal ganglion cells number reflects the severity of disease (based also on PSD value, not included in Medeiros formulas) and well correlate with functional parameters themselves (PSD and VFI). At the same time RGCs number has a good correlation with sectorial thicknesses obtained with OCT: a better correlation with a specific sector could indicate the region from which retinal ganglion cell loss starts, but we are not able to demonstrate it with our results.

The main strength of our study is represented to the potential use of RGC counts for their classification and diagnostic capabilities. Furthermore, other interesting indices, as GLV, could be used instead of RGC counts, whose estimation requires time and data elaboration; in fact GLV showed highest level of correlation (r > −0.8) with RCG counts. On the other hand the not so great number of patients could be surely considered a limit of our study, together with the impossibility to evaluate the measurement variability as explained in “Methods”. Nevertheless, in our study we tried to simulate a daily clinical practice, in which just one reliable test (both SAP and OCT) is performed and evaluated by the ophthalmologist: higher is the number of the tests, smaller will be the compliance of the patient and the reliability of the test itself.

## Conclusions

A very good correlation exists between MD, PSD and VFI and an analogous result can be noticed between RNFL and GCC, both total and sectorial, above all in early glaucoma. This confirm the anatomic relation existing between macular RGCs and their axons and dendrites measured around the optic nerve. Therefore in clinical practice both GCC and RNFL could be used to detect structural changes usually occurring in early glaucoma.

FLV and GLV have a good or very good correlation with ganglion cells counts: in particular, GLV correlates with circumpapillary and macular RGC counts better than FLV and could be used during clinical practice instead of Medeiros’ formula.

RGC counts estimated with Medeiros’ formula is not just an interesting combination of functional and structural parameters, but also a method to summarize in an objective way a wide and partially personal clinical reasoning. Moreover, RGC counts discriminate various stages of disease better than any other parameter singularly considered. Although further studies with a larger number of patients are necessary.

## Additional file

Additional file 1:
**STROBE Statement.** (DOC 89 kb)

## Abbreviations

BCVA: Best-Corrected Visual Acuity
DLS: Differential Light Sensitivity
FD: Fractional Deviation
FLV: Focal Loss Volume
GCC: Ganglion cell complex
GLV: Global Loss Volume
GSS2: Glaucoma Staging System 2
HRT: Heidelberg Retina Tomograph
IOP: Intra-Ocular Pressure
IQR: Interquartile Range
MD: Mean Defect
OCT: Optic Coherence Tomography
ONH: Optic Nerve Head
PD: Pattern Deviation
POAG: Primary Open-Angle Glaucoma
PSD: Pattern Stardard Deviation
RGC: Retinal Ganglion Cells
RNFL: Retinal nerve fiber layer
SAP: Standard Automated Perimetry
SD: Standard Deviation
SITA: Swedish Interactive Threshold Algorithm
SD - OCT: Spectral-Domain Optic Coherence Tomography
VFI: Visual Field Index
