# Supplementary material for: Structure/Function relationship and retinal ganglion cells counts to discriminate glaucomatous damages
Source: BMC Ophthalmol. 2015 Dec 29;15:185. doi: 10.1186/s12886-015-0177-x (PMC4693406; doi:10.1186/s12886-015-0177-x)
Supplement: Additional file 1: — STROBE Statement. (DOC 89 kb) [file 12886_2015_177_MOESM1_ESM.doc]

STROBE Statement—Checklist of items that should be included in reports of ***cross-sectional studies***

|  | Item No | Recommendation |
| --- | --- | --- |
| **Title and abstract** | 1 | (*a*) STRUCTURE/FUNCTION RELATIONSHIP AND RETINAL GANGLION CELLS COUNTS TO DISCRIMINATE GLAUCOMATOUS DAMAGES |
|  |  | (*b*)  Purpose  The aim of the current study is to analyse the correlation between RGC count, estimated by Medeiros’ formula, and the structural and functional parameters in patients examined for glaucoma and to evaluate Standard Automated Perimetry (SAP), Optical Coherence Tomography (OCT) and Retinal Ganglion Cells (RGC) counts capability to discriminate the weight of the disease itself.  Methods  94 eyes of 50 consecutive patients clinically referring to glaucoma service of the University Eye Clinic were submitted to a complete ophthalmic evaluation including SAP and Spectral Domain OCT (SD-OCT) of Retinal Nerve Fiber Layer (RNFL) and macular Ganglion Cell Complex (GCC).  Average thickness of RNFL and macular GCC, parameters Global Loss Volume (GLV) and Focal Loss Volume (FLV) over the entire GCC map were taken into account. Estimates of RGC were obtained with the help of a model already published by Medeiros et al. combining light sensitivities from SAP and retinal thickness from OCT. The RGC count was estimated in the entire visual field (central 24°) and in the GCC macular area and then compared with functional and morphological parameters applying Pearson’s correlation coefficient.  Results  After the classification of the patients by the Glaucoma Staging System 2 of Brusini, we noticed a good correlation among the functional parameters considered, even if the Visual Field Index is unable to identify early glaucoma. An analogous result can be observed for structural data (RNFL and GCC). The correlation detected between functional and structural parameters was moderate. Great differences in RGC counts were found between groups at various stages of glaucoma. GLV showed highest level of correlation (r> - 0.8) with RCG counts.  Conclusions  Estimate circumpapillary and macular RGC counts can discriminate various stages of  the disease and there is also a good/very good correlation with both functional and structural parameters. GLV could be used instead of RGC counts in clinical practice. |
| Introduction | | |
| Background/rationale | 2 | Glaucoma is an optic neuropathy characterized by RGC loss and RNFL injury: this results in functional and morphological changes. The first can be observed by SAP, the second by OCT that measures the RNFL and GCC thicknesses. Nevertheless, diagnosis of early glaucoma may be difficult. Recently, Medeiros et al. derived an empirical formula combining the measurement of structural and functional tests to provide an estimate of RGC. |
| Objectives | 3 | The aim of the current study is to analyse the correlation between RGC count, estimated by Medeiros’ formula, and the structural and functional parameters in patients examined for glaucoma and to evaluate SAP, OCT and RGC counts capability to discriminate the weight of the disease itself. |
| Methods | | |
| Study design | 4 | 94 eyes of 50 consecutive patients clinically referring to glaucoma service of the University Eye Clinic were submitted to a complete ophthalmic evaluation including SAP and Spectral Domain OCT (SD-OCT) of Retinal Nerve Fiber Layer (RNFL) and macular Ganglion Cell Complex (GCC).  Average thickness of RNFL and macular GCC, parameters Global Loss Volume (GLV) and Focal Loss Volume (FLV) over the entire GCC map were taken into account. Estimates of RGC were obtained with the help of a model already published by Medeiros et al. combining light sensitivities from SAP and retinal thickness from OCT. The RGC count was estimated in the entire visual field (central 24°) and in the GCC macular area and then compared with functional and morphological parameters applying Pearson’s correlation coefficient. |
| Setting | 5 | Fifty glaucoma patients or glaucoma suspects (94 eyes) were consecutively recruited at the University Eye Clinic, IRCCS San Matteo Hospital, Pavia, Italy. Each study participant underwent a complete ophthalmic examination including Standard Automated Perimetry (SAP) and Spectral Domain-Optical Coherence Tomography (SD-OCT). |
| Participants | 6 | Primary open-angle glaucoma patients or glaucoma suspects referring to University Eye Clinic were recruited. If best corrected visual acuity (BCVA) was ≥7/10 the patient was included in the study. Participants were excluded if they had any retinal disease, non glaucomatous optic neuropathy or any significant coexisting systemic disease with possible ocular involvement, such as diabetes mellitus. |
| Variables | 7 | Age, Mean Deviation (*MD*), Pattern Standard Deviation (*PSD*), Visual Field Index (*VFI*), Global Loss Volume (*GLV*), Focal Loss Volume (*FLV*), average RNFL thickness (*RNFL av.*), RNFL superior thickness (*RNFL av. sup.*), RNFL inferior thickness (*RNFL av. inf.*), RNFL temporal thickness (*RNFL av. temp.*), GCC average thickness (*GCC av.Total*), GCC superior thickness (*GCC av. sup.)* and GCC inferior thickness (*GCC av. inf.)*. To these, we also added *RGC count* and *RGC count GCC* obtained using Medeiros’ algorithm. |
| Data sources/ measurement | 8* | All data were directly provided by the instruments. Regarding SAP, global indices Mean Defect (MD), Pattern Standard Deviation (PSD) and Visual Field Index (VFI) were taken into account. All patients were well trained to SAP with more than one reliable visual field test in the past. All the visual field considered were reliable with fixation losses <20% and false positive and negative errors <15% and were performed only once on the examination day. Retinal Nerve Fibre Layer (RNFL) and Ganglion Cell Complex (GCC) thicknesses, Global Loss Volume (GLV) and Focal Loss Volume (FLV) were measured by SD-OCT: the first OCT test with a “Good” Scan Quality Index (≥40) was taken into account. |
| Bias | 9 | All OCT imaging were of “good quality”. Scan Quality index was ≥40. All perimetric tests were performed by trained patients and were reliable. |
| Study size | 10 | Sample size considerations  With a sample of about 90 eyes, a coefficient r will be statistically significant (p<0.001) if greater than 0.34 as alternative hypothesis versus null hypothesis of r=0. |
| Quantitative variables | 11 | If quantitative data were normally distributed mean and standard deviation (SD) was used to summarize results; if data were not normally distributed median and interquartile ranges (IQR) was used. For quantitative variables, comparisons among three groups were performed with the ANOVA test or with One-way analysis of variance by ranks (Kruskal-Wallis Test) and post-hoc tests were performed to correct for multiple comparisons. Correlations between quantitative variables were evaluated with Pearson’s *r coefficient*. The strength of correlation was defined as “very poor” (r<20), “poor” (0.21<r<0.40), “moderate” (0.21<r<0.60), “good” (0.61<r<0.80) or “very good” (0.81<r<1). All reported p-values were two-sided. Differences were considered significant when the two-sided p value was <0.01. All analyses were carried out with the STATA software (vers: 13, Stata Corporation, College Station, 2013, Texas, USA). |
| Statistical methods | 12 | If quantitative data were normally distributed mean and standard deviation (SD) was used to summarize results; if data were not normally distributed median and interquartile ranges (IQR) was used. Qualitative data were described as frequencies and percentages. For quantitative variables, comparisons among three groups were performed with the ANOVA test or with One-way analysis of variance by ranks (Kruskal-Wallis Test) and post-hoc tests were performed to correct for multiple comparisons. Chi-square test or Fisher’s exact tests were used to compare qualitative variables. Correlations between quantitative variables were evaluated with Pearson’s *r coefficient*. The strength of correlation was defined as “very poor” (r<20), “poor” (0.21<r<0.40), “moderate” (0.21<r<0.60), “good” (0.61<r<0.80) or “very good” (0.81<r<1). All reported p-values were two-sided. Differences were considered significant when the two-sided p value was <0.01. All analyses were carried out with the STATA software (vers: 13, Stata Corporation, College Station, 2013, Texas, USA). |
| Results | | |
| Participants | 13* | The study included 94 eyes of 50 patients (M/F : 27/23) with SAP and iVue SD-OCT scans available. Six eyes were previously excluded owing to their inability to look fixedly. According to GSS 2, the 94 eyes were classified into stage 0 (39 eyes), borderline (10 eyes), stage 1 (14 eyes), stage 2 (7 eyes), stage 3 (7 eyes), stage 4 (8 eyes) and stage 5 (9 eyes). Three groups were formed: group 0 (stage 0 + borderline; 46 eyes), group 1 (stage 1 + stage 2; 19 eyes) and group 2 (stage 3 + stage 4 + stage 5; 24 eyes). |
|  |  | We considered data concerning a single eye for six of these patients, because of the absence of fixation of the contra lateral eye resulting from advanced glaucoma so that SAP and OCT could not be performed. |
|  |  |  |
| Descriptive data | 14* | All considered patients were Caucasian affected or suspected for Primary Open-Angle Glaucoma. Social differences were not considered. |
|  |  | For each considered patient, every variable of interest was available. |
| Outcome data | 15* | There are statistical significance values of all considered parameters comparing groups 0, 1 and 2. MD and PSD derived from SAP always showed a statistically significant difference (p<0.01) in the comparison between groups. Nevertheless, VFI was not statistically significant (p=0.563) comparing groups 0 and 1. A similar pattern can be observed for circumpapillary and macular structural parameters: both total and sectorial RNFL and GCC thicknesses showed a p>0.01 in comparison between group 0 and group 1, as it can been observed in Table 2. Two exceptions were represented by superior RNFL average and temporal RNFL average (p<0.01): in particular, as it could be seen in Figure 4, temporal RNFL average thickness showed a significant decrease comparing group 0 (98.96 μm; SD 14.17 μm) and group 1 (78.42 μm; SD 15.37 μm). Recorded circumpapillary RGC count (IQR) in group 0 was 935795.4 (155703.3), in group 1 was 680545.1 (149734) while in group 2 was 326780.8 (164051.3). On the other hand, macular RGC count (IQR) was 909314.2 (173798.6) in group 0, 662358.6 (139205.6) in group 1 and 305989.2 (170404.9) in group 2. |
|  |  | The study included 94 eyes of 50 patients (M/F : 27/23) with SAP and iVue SD-OCT scans available. Six eyes were previously excluded owing to their inability to look fixedly. According to GSS 2, the 94 eyes were classified into stage 0 (39 eyes), borderline (10 eyes), stage 1 (14 eyes), stage 2 (7 eyes), stage 3 (7 eyes), stage 4 (8 eyes) and stage 5 (9 eyes). Three groups were formed: group 0 (stage 0 + borderline; 46 eyes), group 1 (stage 1 + stage 2; 19 eyes) and group 2 (stage 3 + stage 4 + stage 5; 24 eyes).  The patients were not uniformly distributed within the different groups : 70 of the 94 eyes were normal or affected by early glaucoma. This sample was representative for an initial glaucomatous damage, which was related to perimetric indices, as expected, but also to morphometric data provided by OCT.  Functional parameters, derived from SAP, showed a significant difference comparing group 0/1 and group 2, but not between group 0 and group 1.  Table 2 showed statistical significance values of all considered parameters comparing groups 0, 1 and 2. MD and PSD derived from SAP always showed a statistically significant difference (p<0.01) in the comparison between groups. Nevertheless, VFI was not statistically significant (p=0.563) comparing groups 0 and 1: this could demonstrate that not all SAP parameters, considered separately, are always so sensitive in discriminating healthy subjects from those with early/moderate perimetric defects. As demonstrated by Brusini's GSS2, these parameters should be considered in couple at least (and not individually) to discriminate various stage of glaucoma.  A similar pattern can be observed for circumpapillary and macular structural parameters: both total and sectorial RNFL and GCC thicknesses showed a p>0.01 in comparison between group 0 and group 1. Two exceptions were represented by superior RNFL average and temporal RNFL average (p<0.01): in particular, as it could be seen in Figure 4, temporal RNFL average thickness showed a significant decrease comparing group 0 (98.96 μm; SD 14.17 μm) and group 1 (78.42 μm; SD 15.37 μm).  On the contrary, estimate of retinal ganglion cells count, obtained applying Medeiros algorithms in circumpapillary and macular regions, seems to be able to discriminate patients at different stages of neuropathy. More in detail, as shown in Figures 5-6, recorded circumpapillary RGC count (IQR) in group 0 was 935795.4 (155703.3), in group 1 was 680545.1 (149734) while in group 2 was 326780.8 (164051.3). On the other hand, macular RGC count (IQR) was 909314.2 (173798.6) in group 0, 662358.6 (139205.6) in group 1 and 305989.2 (170404.9) in group 2 . Comparing these three groups, we always found p<0.01 for both RGC count and RGC count GCC.  Considering Pearson’s correlation coefficient (r), there is no relation between SAP/OCT and the age of patients, so that our results are not influenced by this parameter (Table 3): in fact, no one of the considered parameters showed a statistically significant age-related difference. As expected, Pearson’s coefficient showed a good or very good correlation (r always >0.61) between parameters provided by the same instruments (SAP or OCT), but also between circumpapillary RNFL thickness and the inner macular retina (GCC), both total (r=0.85) and sectorial superior/inferior (r=0.78 and r=0.87 respectively). Nevertheless, there is a substantially moderate correlation between structural and functional parameters, as shown in Table 3. Good and very good correlations (r always ≥0.61) can be observed comparing both functional and structural indices with RGC count both in the central macular area and paracentral one which correspond to the 24-2 visual field.  There is a good inverse correlation between FLV and the two RCG counts (r=-0.79); likewise, GLV is in a very good inverse relation with RGC count (r=-0.81) and RGC count GCC (r=-0.82). |
| Other analyses | 17 | Report other analyses done—eg analyses of subgroups and interactions, and sensitivity analyses. VFI was not statistically significant (p=0.563) comparing groups 0 and 1: this could demonstrate that not all SAP parameters, considered separately, are always so sensitive in discriminating healthy subjects from those with early/moderate perimetric defects. As demonstrated by Brusini's GSS2, these parameters should be considered in couple at least (and not individually) to discriminate various stage of glaucoma. As expected, Pearson’s coefficient showed a good or very good correlation (r always >0.61) between parameters provided by the same instruments (SAP or OCT), but also between circumpapillary RNFL thickness and the inner macular retina (GCC), both total (r=0.85) and sectorial superior/inferior (r=0.78 and r=0.87 respectively) (Table 3). Nevertheless, there is a substantially moderate correlation between structural and functional parameters, as shown in Table 3. Good and very good correlations (r always ≥0.61) can be observed comparing both functional and structural indices with RGC count both in the central macular area and paracentral one which correspond to the 24-2 visual field.  There is a good inverse correlation between FLV and the two RCG counts (r=-0.79); likewise, GLV is in a very good inverse relation with RGC count (r=-0.81) and RGC count GCC (r=-0.82). |
| Discussion | | |
| Key results | 18 | Summarise key results with reference to study objectives. A very good correlation exists between MD, PSD and VFI and an analogous result can be noticed between RNFL and GCC, both total and sectorial, above all in early glaucoma. FLV and GLV have a good or very good correlation with ganglion cells counts: in particular, GLV correlates with circumpapillary and macular RGC counts better than FLV and could be used during clinical practice instead of Medeiros’ formula.  RGC counts estimated with Medeiros’ formula is not just an interesting combination of functional and structural parameters, but also a method to summarize in an objective way a wide and partially personal clinical reasoning. Moreover, RGC counts discriminate various stages of disease better than any other parameter singularly considered. Although further studies with a larger number of patients are necessary. |
| Limitations | 19 | The not so great number of patients could be surely considered a limit of our study, together with the impossibility to evaluate the measurement variability. Nevertheless, in our study we tried to simulate a daily clinical practice, in which just one reliable test (both SAP and OCT) is performed and evaluated by the ophthalmologist: higher is the number of the tests, smaller will be the compliance of the patient and the reliability of the test itself. |
| Interpretation | 20 | The very good correlation between functional/structural parameters confirm the anatomic relation existing between macular RGCs and their axons and dendrites measured around the optic nerve. Therefore in clinical practice both GCC and RNFL could be used to detect structural changes usually occurring in early glaucoma. Moreover, RGC counts discriminate various stages of disease better than any other parameter singularly considered. Although further studies with a larger number of patients are necessary. |
| Generalisability | 21 | The main strength of our study is represented to the potential use of RGC counts for their classification and diagnostic capabilities. Furthermore, other interesting indices, as GLV, could be used instead of RGC counts, whose estimation requires time and data elaboration; in fact GLV showed highest level of correlation (r> -0.8) with RCG counts. |
| Other information | | |
| Funding | 22 | 1. Medeiros FA, Weinreb RN et al. Estimating the rate of retinal ganglion cell loss in glaucoma. Am J Ophthalmol 2012; 154:814-824 |

*Give information separately for exposed and unexposed groups.

**Note:** An Explanation and Elaboration article discusses each checklist item and gives methodological background and published examples of transparent reporting. The STROBE checklist is best used in conjunction with this article (freely available on the Web sites of PLoS Medicine at http://www.plosmedicine.org/, Annals of Internal Medicine at http://www.annals.org/, and Epidemiology at http://www.epidem.com/). Information on the STROBE Initiative is available at www.strobe-statement.org.
